# Macrophage efferocytosis with VEGFC and lymphangiogenesis: rescuing the broken heart

**DOI:** 10.1172/JCI158703

**Published:** 2022-05-02

**Authors:** Patricia A. D’Amore, Pilar Alcaide

**Affiliations:** 1 Schepens Eye Research Institute of Mass Eye and Ear, Departments of Ophthalmology and Pathology, Harvard Medical School, Boston, Massachusetts, USA.; 2 Department of Immunology, Tufts University School of Medicine, Boston, Massachusetts, USA.

## Abstract

Cardiac repair following ischemic injury is indispensable for survival and requires a coordinated cellular response involving the mobilization of immune cells from the secondary lymphoid organs to the site of damage. Efferocytosis, the engulfment of cell debris and dying cells by innate immune cells, along with lymphangiogenesis, the formation of new lymphatic vessels, are emerging as central to the cardiac healing response. In this issue of the *JCI*, Glinton et al. used state-of-the-art approaches to demonstrate that efferocytosis induced vascular endothelial growth factor C (VEGFC) in myeloid cells and stimulated lymphangiogenesis and cardiac repair. These findings provide impactful mechanistic information that can be leveraged to therapeutically target pathways in cardiac repair and ischemic heart failure.

## Innate immunity and the lymphatic system

Ischemic heart disease is estimated to affect over 120 million people worldwide. Immune and fibrotic responses work in tandem to repair the heart after ischemia by physically stabilizing the infarct and removing cell debris through efferocytosis. However, the same fibrotic scar that is critical for preventing cardiac rupture and death early on contributes to later systolic dysfunction and chronic heart failure (HF). A better understanding of the coordinated healing response is necessary to discover improved therapeutics for ischemic heart disease.

The immune response to ischemic myocardial injury mobilizes innate and adaptive immune cells to the site of injury (1). Efferocytosis is one mechanism by which myocardial macrophages coordinate cardiac repair by simultaneously clearing cell debris, taking up cardiac antigens, and trafficking to the lymph nodes where macrophages enhance the adaptive immune response necessary for myocardial healing (2). These steps position the immune response and the lymphatic system at the epicenter of cardiac repair. While the role of cardiac lymphatics has been extensively studied during cardiac development, its role in cardiac repair is only recently emerging (3, 4). Since defective efferocytosis leads to accelerated HF in mice, understanding the crosstalk between efferocytosis and lymphatics is an exciting avenue of investigation that may provide insight into the pathogenic processes underlying chronic ischemic HF (5–7).

The vascular endothelial growth factor (VEGF) family in mammals is composed of VEGFA, placental growth factor, VEGFB, VEGFC, and VEGFD, which signal though VEGFR1 (also known as FLT), VEGFR2 (also known as KDR or FLK1), and VEGFR3 (also known as FLT4). Most relevant to the current discussion, genetic knockout of *Vegfc* is embryonically lethal because of the lack of lymphatics (8), and the binding of VEGFC to VEGFR3 on lymphatic endothelial cells induces lymphangiogenesis (9). Macrophage production of VEGFC by CD11b^+^ myeloid cells has been reported in the inflamed skin of mice (10), and intramyocardial administration of VEGFC to mice had immunomodulatory and proangiogenic effects after MI, resulting in improved cardiac function (11).

**Figure 1:**
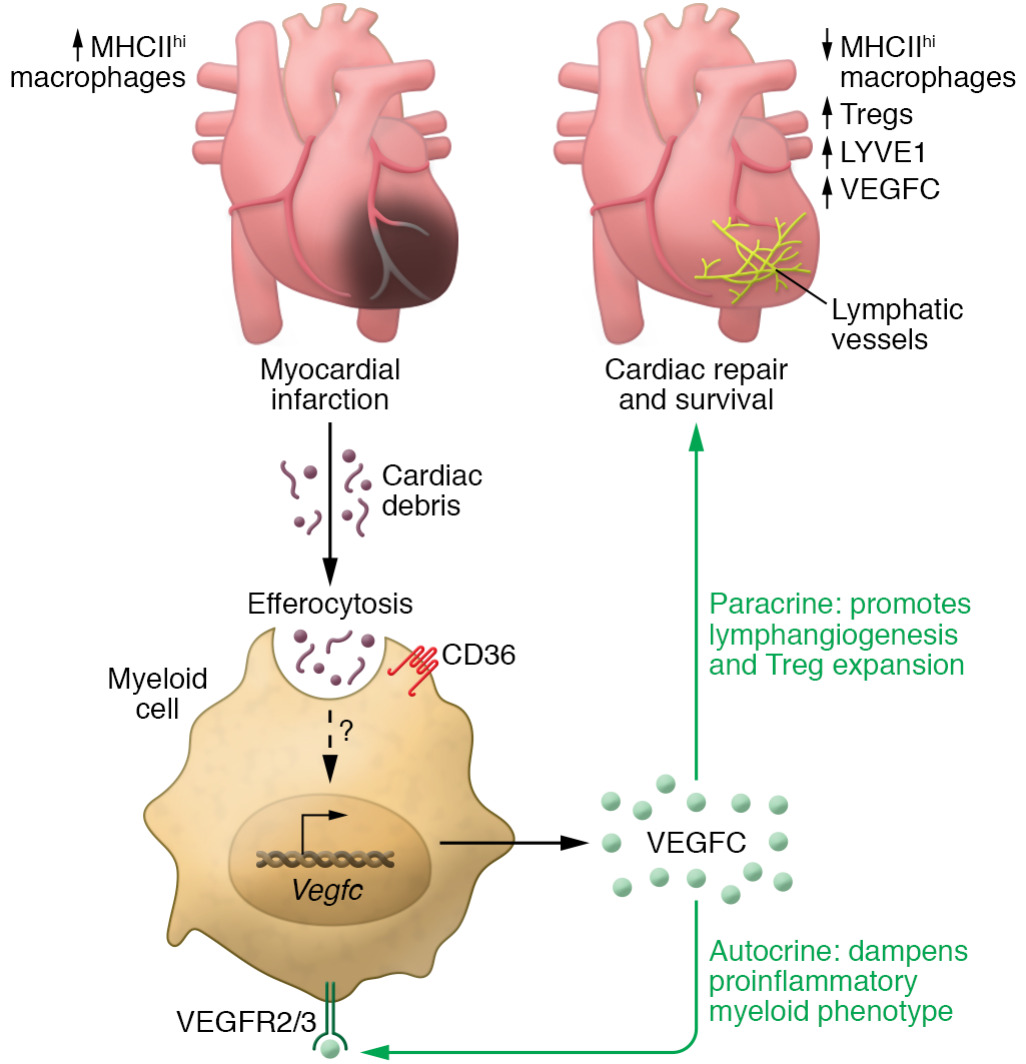
Macrophage efferocytosis induces VEGFC release, leading to lymphangiogenesis, and contributes to cardiac repair after ischemia. CD36-dependent efferocytosis induces VEGFC release from myeloid cells and activates macrophage expression of VEGFC through as-yet unknown mechanisms. VEGFC functions in an autocrine manner to dampen macrophage proinflammatory cytokine production as well as in a paracrine fashion to induce lymphangiogenesis and modulate the presence of immune cells, such as Tregs and myeloid cells expressing low levels of MHCII, that are necessary to resolve inflammation and promote myocardial repair.

In this issue of the *JCI*, Glinton et al. (12) hypothesized that efferocytosis triggers myeloid VEGFC production, which, in turn, promotes the lymphangiogenic response in cardiac repair, thus positioning myeloid-derived VEGFC in the midst of the complex interplay between the inflammatory and lymphangiogenic responses to myocardial ischemia (refs. 4, 13 and Figure 1). The authors used a variety of strategies and models that included tracking myocyte uptake by macrophages using myosin heavy-chain reporter mice and myeloid-specific scavenger receptor CD36-deficient mice (*Cd36^–/–^*). They initially observed that cardiac antigen accumulation in myeloid cells in the lymph nodes after MI, concomitant with increased myocardial expression of VEGFC and the lymphatic endothelial cell marker lymphatic vessel endothelial hyaluronan receptor 1 (LYVE1). Cell-intrinsic evidence that efferocytosis induced VEGFC was provided in vitro, as apoptotic cells added to primary cardiac macrophages resulted in elevated *Vegfc* mRNA levels and VEGFC secretion. *Cd36^–/–^* mice showed reduced myeloid accumulation of cardiac antigen in the lymph nodes, a striking reduction of myocardial LYVE1 and VEGFC expression, and decreased cardiac macrophage VEGFC expression compared with control mice, further supporting the notion that VEGFC expression after MI depends on myeloid CD36. These results additionally suggest that macrophage-derived VEGFC induces lymphangiogenesis downstream of efferocytosis (12).

## VEGFC signaling and cardiac lymphatics in cardiac repair

Glinton and colleagues also used elegant *Vegfc* gain- and loss-of-function approaches in vivo to demonstrate that *Vegfc* deficiency in myeloid cells resulted in reduced lymphatic cell density, increased infarct size, and exaggerated left ventricular remodeling after MI. Conversely, *Vegfc* overexpression in myeloid cells stimulated lymphangiogenesis and improved systolic function. The authors went one step further to investigate the clinical importance of these findings. Using ischemia reperfusion (I/R) in mice, they found that LYVE1 expression was increased in myocardial lymphatics at the post-I/R risk area and that macrophage *Vegfc* was required for improved cardiac function after reperfusion. These studies confirm a central role for myeloid-derived VEGFC in lymphangiogenesis and cardiac repair with consequences on systolic function (12).

Glinton and colleagues considered the complexity of the immune, fibrotic, and angiogenic responses in the heart following ischemia and investigated the role of myeloid VEGFC in the immune response during cardiac repair. They combined RNA-Seq and flow cytometry of cardiac tissue to characterize the immune landscape, discovering that myeloid deficiency of *Vegfc* resulted in increased proinflammatory cytokine and chemokine expression, along with increased numbers of innate immune cells seven days after MI. Although similar macrophage quantities were found in mice that overexpressed *Vegfc*, in myeloid cells, a higher proportion of macrophages displayed low MHC class II (MHCII) expression, accounting for reduced myocardial inflammation seven days after MI. Conversely, *Cd36^–/–^* mice showed a higher fraction of myocardial macrophages expressing high levels of MHCII, supporting the notion that efferocytosis is upstream of these signals. In line with myeloid-derived VEGFC regulating adaptive immunity through myeloid MHCII expression, Glinton et al. (12) also found that lower numbers of Tregs in myeloid *Vegfc^–/–^* mice were required for post-MI cardiac repair (14, 15) compared with control mice. These findings were consistent with the idea that myeloid VEGFC promotes inflammation resolution. Macrophages isolated from *Vegfc^–/–^* mice had elevated *Tnfa*, *Il6*, and *Il12* gene expression that could be reduced with administration of VEGFC. In addition to the paracrine function of macrophage-produced VEGFC modulating cardiac lymphangiogenesis and immune function, these observations support an autocrine function for VEGFC through VEGFR3 to suppress excessive secretion of proinflammatory cytokines. This possibility was partially corroborated in vivo with the pharmacological inhibition of VEGFR3 using MAZ51, which reduced cardiac antigen uptake by macrophages (12). Studies in myeloid-specific *Vegfr3*-deficient mice could determine whether this autocrine proinflammatory pathway in macrophages accounts for the observed protection of MAZ51.

## Concluding remarks

Glinton et al. (12) provide mechanistic insights into a role for myeloid-derived VEGFC in cardiac repair, functioning through macrophage-intrinsic and -extrinsic functions. Perhaps the most important aspects of this study are the identification of efferocytosis as a trigger for the induction of VEGFC in macrophages and that the presence of VEGFC itself directs the inflammatory and resolution stage of the macrophage. The underlying mechanisms for the reported protective effect of myeloid VEGFC are therefore two-fold: enhancement of lymphangiogenesis, which is central to improved post-MI cardiac function (16), and immunomodulation of innate and adaptive immune responses during cardiac repair. It remains to be determined whether other efferocytosis-triggered angiogenic factors besides VEGFC, such as VEGFA (5), promote lymphangiogenesis and vascular angiogenesis after ischemia.

In addition, the specific molecular signals downstream of CD36 that lead to *Vegfc* transcription and release remain unknown. CD36 is a fatty acid transporter, and while Glinton et al. (12) used inhibitors of fatty acid oxidation to demonstrate that efferocytosis depends on CD36 in VEGFC production in mice, the role for CD36 remained independent of fatty acid oxidation. It is possible that fatty acid oxidation contributes to lymphangiogenesis in humans with hyperlipidemia and obesity after MI. Therefore, similar studies conducted in the context of hyperlipidemia will expand the mechanisms described in the work by Glinton et al. (12).

Although the contribution of cardiac lymphangiogenesis to improved post-MI cardiovascular outcomes is evident (6) and supported by studies that delivered intramyocardial VEGFC (13) and by Glinton et al. (12), a recent study showed that the loss of cardiac lymphatics is not detrimental to heart function after MI (17). The investigation used lymphatic endothelial cell–specific, VE-cadherin–deficient mice, which, despite showing impaired cardiac lymphatic transport and mild myocardial edema, do not exhibit post-MI cardiac dysfunction. The mice also possess an apparently intact cardiac lymphatic network (17). Thus, it will be of interest to explore whether VE-cadherin expression by lymphatic vessels contributes to the maintenance of angiogenic signaling that can be exploited for sustaining maximal lymphangiogenesis during cardiac repair. It is also relevant to examine the connection between efferocytosis and lymphatic VE-cadherin expression after MI. Glinton et al. (12) remind us that macrophages are important early players in cardiac repair, not only via their proinflammatory and proresolution functions that coordinate immune responses, but also by driving lymphangiogenesis through VEGFC and the lymphatic cardiac vasculature that are critical for survival.

## Version 1 05/02/2022

Electronic publication
